# The Role of Diet in Influencing the Diversity of Gut Microbiome Related to Lupus Disease Activities: A Systematic Review

**DOI:** 10.1155/2022/6908677

**Published:** 2022-12-31

**Authors:** Prisly Z. Putri, Laniyati Hamijoyo, Edhyana Sahiratmadja

**Affiliations:** ^1^Faculty of Medicine, Universitas Padjadjaran, Bandung 40161, Indonesia; ^2^Rheumatology Division, Department of Internal Medicine, Faculty of Medicine, Universitas Padjadjaran, Dr. Hasan Sadikin General Hospital, Bandung 40161, Indonesia; ^3^Immunology Study Center, Faculty of Medicine, Universitas Padjadjaran, Bandung 40161, Indonesia; ^4^Department of Biomedical Sciences, Faculty of Medicine, Universitas Padjadjaran, Bandung 40161, Indonesia

## Abstract

Gut microbiome dysbiosis can affect the host immune system. The balance and activity of the gut microbiome, which are influenced by daily diet, might be associated with disease activity in systemic lupus erythematosus (SLE). Therefore, we conducted a systematic review based on the PRISMA guideline to explore the role and types of diet that affects the gut microbiome related to changes in SLE disease activity. All original and full-text English articles in the last ten years were included using predefined keywords according to PEO (population, exposure, and outcome) design in PubMed. The study subjects were carefully analyzed, including lupus-susceptible mice and humans with SLE on various diets. Children and pregnant women populations were excluded. Of 134 studies found, only seven full-text articles had met the inclusion criteria of which only one study conducted in human. This human study showed that dietary polyphenol as dihydrochalcones and flavanones affected the gut microbiome and ameliorated lupus disease activity. On the contrary, dietary flavones increased *Blautia* (family: *Lachnospiraceae*), and that often found in active lupus diseases. Furthermore, six studies in lupus-prone mice models showed that resistant starch (RS), retinoic acid (RA) or all-trans retinoic acid (tRA), and acidic water (AW) had influenced the gut microbiome, leading to an improved lupus development. Conversely, the 2018 commercial rodent diet, vitamin A-retinoic acid (VARA), neutral water (NW), and high tryptophan diet had impacted various microbiomes, resulting in increased lupus activity. Interestingly, several diets have the effect of either increasing or decreasing lupus disease activity depending on the microbiome they affect, such as AW associated with *Turicibacter* spp., which is frequently found in active lupus disease, and tRA in *Bacteroidetes* associated with renal pathology. To conclude, diet can influence the gut microbiome, which is related to the disease activity process of SLE.

## 1. Introduction

Systemic lupus erythematosus (SLE) is an autoimmune disease resulting from a loss of immune tolerance. About 0.3 to 23.7 per 100,000 individuals globally have been diagnosed with SLE yearly. The prevalence varies from 6.5 to 178.0 per 100,000 population, predominantly in productive-age women and more frequent in adults than children [1, 2].

SLE is a unique disease with various atypical manifestations, making it challenging to recognize, prevent, and treat [1, 3]. On the other hand, the pathogenesis of SLE is still unclear and multifactorial [4]. The environmental triggers play an important role in SLE development in genetically susceptible individuals, including hormones (e.g., prolactin and estrogen), drugs (e.g., oral contraceptive and postmenopausal hormone therapy), UV light, infections (e.g., cytomegalovirus and Ebstein–Barr virus), toxins (e.g., silica dust), and cigarette smoking, as well as dietary factors and gut microbiota [5, 6].

In the last 20 years, many studies on a diet have been associated with SLE and offer a promising alternative treatment as it has many advantages in improving the quality of life to the potential for prophylactic effects [7–10]. Nowadays, the microbiome is a hot topic in research as it influences the physiology or pathology of the host. Dysbiosis or imbalance of the microbiome is a variation of the gut microbial composition in the shape of loss of beneficial organisms and overgrowth of harmful organisms, leading to loss of microbial diversity. Dysbiosis can affect the regulation of the development and function of the immune system, which may play a role in the development of autoimmune diseases such as SLE [11]. Further dysbiosis can damage the intestinal barrier by penetration of living bacteria or removal of dead bacterial components outside the intestine [12, 13]. A study on childbearing-age female lupus-prone mice has shown an increase in *Lachnospiraceae* and a decrease in *Lactobacilli* [14]. Of note, the prevalence of human SLE is predominated (99%) in female in their child-bearing age; therefore, microbiome disbalance plays a role as a risk factor in SLE development. At the microbiome level, dietary factors are determined by several factors, among others, the type of diet.

At the microbiome level, dietary factors are determined by several factors, among others, the type of diet. One of the roles of diets' pattern or types is its effect on the formation of inflammatory or anti-inflammatory metabolites [15]. The correct diet in SLE patients can improve body homeostasis, prevent medication side effects, increase the period of remission, and improve physical and mental well-being [9]. For example, high fiber in the Mediterranean diet has been shown to reduce the risk of CVD and inflammatory markers, as well as to improve anthropometric profiles in SLE patients [16–19]. Clinical complication and comorbidities resulting from SLE therapy, can also be controlled with a diet; for example, diet with high vitamins, minerals, monounsaturated fatty acid (MUFA), or polyunsaturated fatty acid (PUFA) with moderate energy consumption [20, 21]. Inappropriate dietary intake shows worsening SLE disease activity due to changes in the microbiome's composition through increased inflammation and the effect of its antigen [14]. A Western diet low in fiber, high in sugar and fat, and rich in processed foods can worsen SLE activity by altering the diversity of the gut microbiome and disrupting the intestinal barrier [22, 23]. Furthermore, the influence of SLE treatment on gut microbiome might also play a pivotal role, such as the use of steroids, immunosuppressive drugs, and hydroxychloroquine. The precise mechanism by which diet influences the SLE disease process is still not fully elucidated, and there is little literature relating to the influence of diet on the gut microbiome. Therefore, this review has explored and discussed in more detail the type and the role of diet in influencing the gut microbiome associated with SLE disease activity. The results of this study might provide information for future research in nutritional interventions for SLE patients.

## 2. Materials and Methods

### 2.1. Search Strategy and Study Selection

This systematic review was conducted according to the PRISMA guideline. The literature source search was carried out using electronic data of PubMed until the end of August 2021. The keyword was searched according to PEO (population, exposure, and outcome) design; the population was the microbiome in patients with systemic lupus erythematosus, the exposure refers to dietary factors, and the outcome was in the form of SLE disease activity. The keywords had been linked using the Boolean operator, as follows: (((diet) OR (dietary factor) OR (nutrition) OR (dietary intervention) OR (carbohydrate) OR (protein) OR (vitamin) OR (fiber) OR (mineral)) AND ((microbiome) OR (gut microbiome) OR (gut microbiota) OR (microbiota)) AND ((lupus) OR (SLE) OR (systemic lupus erythematosus) OR (human lupus))). In addition, we included all original full-text research articles in English within the last ten years, and as subjects were human and mice models of SLE.

Interventions in the form of diets that influence the gut microbiome targeting changes in SLE disease activity were included with no restrictions, especially on types of diet. Outcomes were measured by the changes in the gut microbiome and identified changes in the SLE disease process. A beneficial effect existed when dietary intake affecting the gut microbiome might ameliorate lupus disease activity. On the other hand, an adverse effect occurred when diet influencing the gut microbiome worsens SLE activity. The exclusion criteria of the articles were children and pregnant women. Articles found with relevant titles were reviewed for abstracts, and then a full-text review was conducted.

### 2.2. Data Extraction and Quality Assessment

The full texts from included articles were extracted into a table, consisting of collected various data on authors, year, research design, population, exposure, target phyla/genera/families/other, indicators for assessing SLE activity, and study results. The assessment of the quality of the articles was carried out using Joanna Briggs Institute's critical appraisal tools from the Faculty of Health and Medical Sciences of the University of Adelaide to determine the clarity and focus of the discussion on the research, the validity of the methods used, and the importance and usefulness of the research result [24]. Several questions were given that had to be filled in with “Yes,” “No,” “Unclear,” or “Not applicable” answers.

## 3. Results

### 3.1. Study Selection

**Figure 1:**
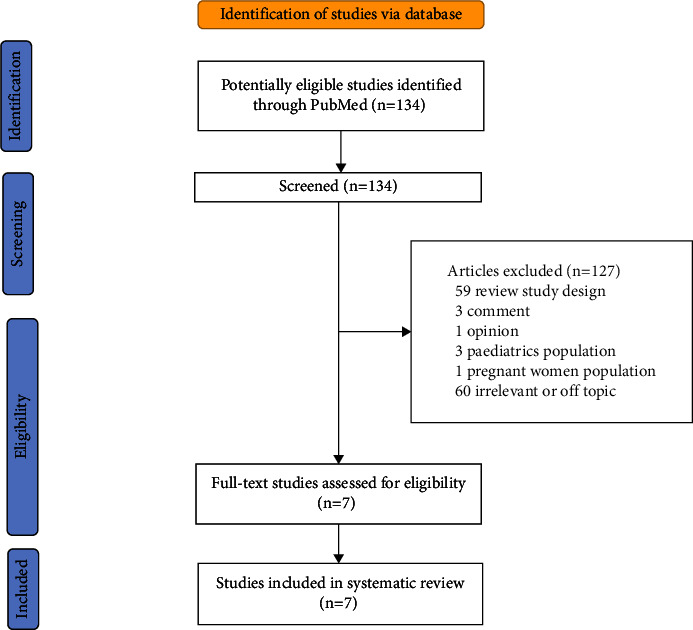
PRISMA flow diagram of study selection.

A qualitative synthesis was conducted with a critical appraisal first. In total, 134 articles were found in the PubMed database. However, only seven articles were relevant to our study, including six experimental studies using lupus-prone mice models and only 1 study using human SLE (Figure 1) [14, 25–30]. The other articles (*n* = 127) were excluded due to review study design (*n* = 59), comment articles (*n* = 3), opinion (*n* = 1), paediatric population (*n* = 3), and pregnant women population (*n* = 1). The other articles (*n* = 60) were irrelevant or off-topic because they did not answer research questions, discussed other diseases, or used other terminology of lupus related to wolf or *Canis lupus familiaris*.

### 3.2. Study Characteristics and Outcomes

The only study with human SLE found in the literature search was a case-control study, including female SLE patients without active disease at the time of sampling. Furthermore, they were not taking antibiotics, glucocorticoids, immunosuppressive drugs, monoclonal antibodies, or other immunotherapy. Additionally, antimalarial drugs and non-steroidal anti-inflammatory drugs were used regularly by 18 of the 20 participants [25].

Furthermore, various types of lupus mice models had been used in experimental studies, such as MRL/Mp-Faslpr (MRL/lpr) mice, pristane-treated Balb/c mice, lupus-prone TLR7.1Tg C57BL/6 (TLR7.1Tg) mice, SWR X NZB F1 (SNF1) mice, and triple congenic lupus-prone mice (B6.Sle1.Sle2.Sle3) stimulated (TC mice), of which five studies considered female mice [26–30], and one study was on both genders [14]. All lupus mice models were not treated for SLE.

Variations of diet composition in this study focused on polyphenols (dihydrochalcones, flavanones, and flavone), vitamin A (RA/tRA and VARA), resistant starch (RS), high tryptophan diet, pH water (acidic water/AW and neutral water/NW), and 2018 commercial rodent diet or isoflavone-richsoy-based diet (2018 diet). Due to dietary intervention, gut microbiota had changed significantly in composition, quality, and quantity. Microbiota was analyzed in all studies, using DNA taken from fecal or gut samples. The main characteristics of the studies about diet that affect the gut microbiome related to SLE disease activity are summarized in Table 1.

**Figure 2:**
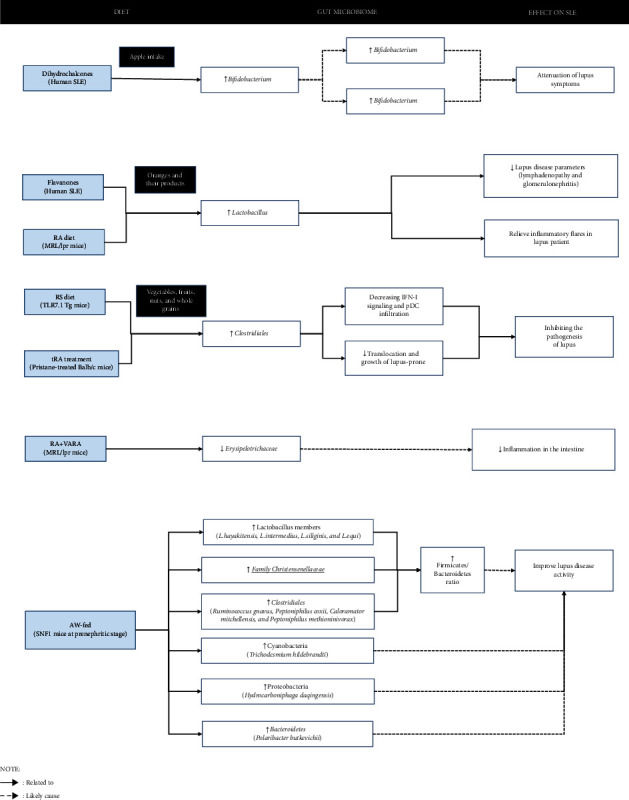
Emerging evidence points to a potential link between diet, gut microbiome, and beneficial effect to SLE.

A beneficial effect was demonstrated in human SLE by dihydrochalcones with *Bifidobacterium*, as well as flavanones in human SLE and RA in MRL/lpr mice on *Lactobacillus*. Moreover, RS diet in TLR7.1Tg and tRA treatment in pristine-treated lupus mice also had a beneficial effect on *Clostridiales*. Furthermore, RA and VARA diets had a beneficial effect on *Erysipelotrichaceae* in MRL/lpr mice. The AW-fed SNF1 mice at the prenephritic stage had a beneficial effect on Lactobacillus members (*L. hayakitensis, L. intermedius, L. siliginis*, and *L. equi*), *Christensenellaceae*, *Clostridiales* (*Ruminococcus gnavus, Peptoniphilus coxii, Caloramator mitchellensis*, and *Peptoniphilus methioninivorax*), *Cyanobacteria* (*Trichodesmium hildebrandti*)*, Proteobacteria* (*Hydrocarboniphaga daqingensis*), and *Bacteroidetes* (*Polaribacter butkevichii*) (Figure 2).

**Figure 3:**
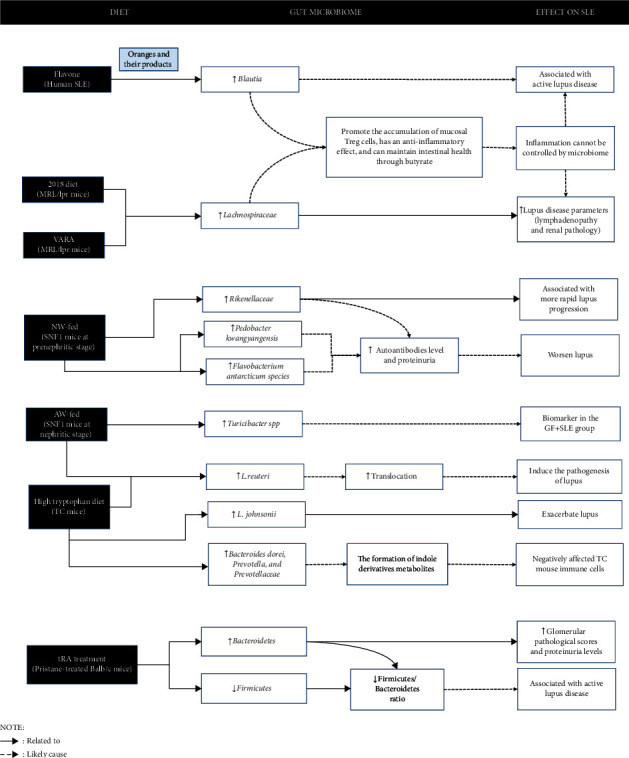
Emerging evidence points to a potential link between diet, gut microbiome, and worsening effect of SLE.

An adverse effect occurred when diet affected the gut microbiome, resulting in increased SLE activity as indicated by the intake of polyphenols in the form of flavones in SLE patients that affect *Blautia*. In SLE mice models, the VARA and 2018 diet had an adverse effect on *Lachnospiraceae* in MRL/lpr mice. Interestingly, VARA in MRL/lpr mice and SNF1 mice fed NW at the prenephritic stage had an adverse effect on *Rikenellaceae*. The SNF1 mice fed NW also had an adverse effect on *Pedobacter kwangyangensis* and *Flavobacterium antarcticum* at the prenephritic stage. Also, AW-fed SNF1 mice in the nephritic stage on *Turicibacter* spp. and *L. reuteri*. Moreover, high tryptophan diet in TC mice had an adverse effect on *L. reuteri*, *L. johnsonii*, *Bacteroides dorei*, *Prevotella*, and *Prevotellaceae*, and tRA diet in Balb/c mice treated with pristane-affected *Firmicutes*/*Bacteroidetes* ratio (Figure 3).

## 4. Discussion

Only a few studies, seven qualified in this systematic review, have been identified to fill in the gaps, since the relationship between the diet-gutmicrobiome-autoimmune diseases is relatively recent. This systematic review has proven that certain diets may influence the microbial profile and affect disease activity in SLE patients or experimental lupus-prone mice. Diet and immunity have complex interrelationships. Nutritional needs and metabolism, as well as physiological responses to food, are influenced by immunity [15]. The microbiome, designated as microorganisms and their ecosystems in the intestine, must be in a balanced number and location. Inflammation or autoimmune disease development can occur when the balance is not reached [12].

### 4.1. Diet That Affects the Gut Microbiome and Its Association with Ameliorating SLE Disease Activity

In SLE, dysbiosis occurs as loss of beneficial organisms, overgrowth of harmful organisms, and loss of microbial diversity [31]. Intake of polyphenols with dihydrochalcones from the regular diet, exclusively apple, in female SLE patients is positively associated with *Bifidobacterium* [25]. This genus has anti-inflammatory properties, and one of its strains, *B. infantis*, has an immunoregulatory effect on lymphocytes, epithelial cells, and dendritic cells [32]. In SLE patients, *Bifidobacterium* can maintain the balance of Tregs, Th17, and Th1 as well as prevent the overactivation of CD4+ lymphocytes [33]. *Bifidobacterium*, an SCFA-producing bacterium, as a probiotic, can maintain the gut and the balance of gastrointestinal microbiota through changes in intestinal pH, produce antimicrobial substances, compete for nutrients, and reduce intestinal lipopolysaccharide levels [33, 34].

Diet with flavanones (mainly from oranges and their products) in SLE patients and RA diet in MRL/lpr mice have shown a positive relationship with *Lactobacillus*, which was decreased in MRL/lpr lupus-prone mice. This relationship negatively correlates with lupus disease parameters (lymphadenopathy and glomerulonephritis) and relieves inflammatory flares in lupus patients [14, 25]. This protective mechanism, produced by lactic acid-producing bacteria, *Lactobacilli*, may occur due to increased immunoregulation by dendritic cells, such as increased Treg balance costimulatory molecules [35]. In addition, there is an increase in the production of anti-inflammatory cytokines (IL-10) which can inhibit the production of IFN-*γ*-induced autoantibodies and clinical nephritis [35, 36].

A decrease in *Clostridiales* (class: Clostridia; phylum: Firmicutes) occurred in TLR7.1Tg lupus-prone mice and could be increased by resistant starch (RS) diet [26]. *Clostridiales* can ferment RS into short-chain fatty acids (SCFAs), thereby inhibiting the pathogenesis of lupus by decreasing IFN-I signalling and pDC. SCFAs also improve the integrity of the epithelial barrier, thereby reducing translocation and growth of lupus-prone bacteria such as *L. reuteri,* especially in mesenteric lymph node complex (MLN), liver, and spleen [26, 37]. Fermentation sites mainly occur in the distal jejunum, cecum, and proximal colon with SCFA metabolites such as acetate, propionate, and butyrate [38].

Resistant starch (RS) is a type of fiber that can suppress lupus-related pathogenesis and mortality and consists of a carbohydrate source in the form of plant oligosaccharides and polysaccharides that cannot be digested in the small intestine [26]. Dietary patterns associated with high fiber intakes, such as vegetables, fruit, and whole grains, have been found in the Mediterranean diet (Med Diet) [16, 39]. A good prognosis is shown in SLE patients with Med Diet, as evidenced by an excellent anthropometric profile, a reduced risk of CVD, and inflammatory markers. The decrease in SLEDAI scores was also shown to be associated with the consumption of fruits, vegetables, olive oil, sofrito, nuts, fish, and nuts, as well as the abstinence from red meat, meat products, sweet foods, and pastries in the Med Diet component, enabling the contribution of the gut microbiota [16].

An increase in the order *Clostridiales* and the genus *Clostridium* has been demonstrated by pristane-induced lupus mice, given tRA (all-trans-retinoic acid) at week two after pristane injection. Although the administration of tRA in the progressive phase of lupus can increase the infiltration of inflammatory cells in the tissues, it shows the immunosuppressant function in the kidneys by reducing glomerulonephritis [29].

The presence of the *Erysipelotrichaceae* family, which belongs to the *Firmicutes* phylum, in MRL/lpr lupus mice depends on gender. There is a decrease in female mice, inversely proportional to male mice [14]. Interestingly, this *Erysipelotrichaceae* family is increased significantly in female SLE patients [40]. Treatment using vitamin A in the form of RA and VARA can lower the level of *Erysipelotrichaceae* [14]. Furthermore, increased inflammation in the intestine is directly proportional to *Erysipelotrichaceae* [41].

In the prenephritic 12-week-old SNF1 mice, AW recipients can enhance several microbiomes such as members of *Lactobacillus* (*L. hayakitensis, L. intermedius, L. siliginis*, and *L. equi*), *Christensenellaceae*, and *Clostridiales* such as *Ruminococcus gnavus*, *Peptoniphilus coxii*, *Caloramator mitchellensis*, and *Peptoniphilus methioninivorax*. These changes have implications for the increase in the *Firmicutes* phylum and contribute to improving the *Firmicutes*/*Bacteroidetes* ratio previously decreased in SLE.

Several phyla, such as cyanobacteria member *Trichodesmium hildebrandti*, *Proteobacteria* member *Hydrocarboniphaga daqingensis*, and *Bacteroidetes* member *Polaribacter butkevichii*, are also increased in AW-fed prenephritic stage SNF1 mice [30]. Although the mechanism of involvement of the gut microbiome is not precisely known, the rate of disease progression such as nephritis severity (measured by grading proteinuria and proinflammatory cytokines), are lower levels in AW recipients than in NW-recipients mice. The production of autoantibodies against DNA (e.g., total serum IgG anti-dsDNA, IgG2a, and IgM) or against nucleohistone complex (e.g., IgG2 and IgM) are at lower levels in AW recipients than in recipient NW, the potential for immune cell infiltration and glomerular necrosis is also low.

### 4.2. Diet Influences the Gut Microbiome and Is Associated with Worsening Disease Activity in SLE

The higher levels of *Lachnospiraceae* are a characteristic of gut microbiota changes in lupus-susceptible MRL/lpr and B6/lpr mice at specific points during lupus progression. In female SLE patients, genus *Blautia* (family: *Lachnospiraceae*) was positively associated with flavone intake, of which the main contributors were oranges and their products. However, flavone-rich foods must be present in a combined whole diet to predict the proportion of *Blautia* [25]. Studies in SLE patients with active lupus disease have shown increased levels of *Blautia* [43].

Retinol mixed with retinyl palmitate, VARA, significantly increased levels of *Lachnospiraceae* and correspondingly increased lupus disease parameters such as lymphadenopathy and renal pathology in MRL/lpr mice [14]. The 2018 commercial rodent diet, which contains phytoestrogenic isoflavone-richsoy-based chow diet, also increases *Lachnospiraceae* related to glomerulonephritis and more severe lupus disease in MRL/lpr mice [28]. Phytoestrogens are estrogen-like substances of plant origin and can produce either good or bad effects on the development of SLE, depending on the estrogen receptor they affect [44]. Proinflammatory effect is associated with increased estrogen receptor alpha (ER*α*) in lupus-prone models [45]. The difference in fiber sources between the 2018 diet with RD diet and 7013 diets is thought to be the cause of the difference in the abundance of *Lachnospiraceae*, where the 2018 diet used a mixture of soluble and insoluble fiber derived from plants. [28].

As part of the XIVa cluster of Clostridia, *Lachnospiraceae* (including *Blautia*) can promote the accumulation of mucosal Treg cells, anti-inflammatory, and maintain intestinal health through butyrate [46]. However, Fas^lpr^ mutations can lead to impaired butyrate-associated T cell apoptosis, so the inflammation in MRL/lpr and B6/lpr mice cannot be controlled by *Lachnospiraceae*, leading to increased SLE disease activity [14, 47].

Another lupus-enriched family in MRL/lpr mice was Rikenellaceae (phylum: *Bacteroidetes*), further enhanced by the VARA diet and associated with more rapid lupus progression [14]. This was also demonstrated by NW in the prenephritic stage of SNF1 mice [30]. An increase in *Pedobacter kwangyangensis* and *Flavobacterium antarcticum* species (phylum: *Bacteroidetes*) has been shown at the prenephritic stage of NW-recipient SNF1 mice [30]. A significant increase in the phylum *Bacteroidetes* that contributes to a decrease in the *Firmicutes*/*Bacteroidetes* ratio is associated with several inflammatory diseases [48, 49]. In autoimmune diseases, the development of inflammation and the outcome of the disease can be influenced by the balance of microorganisms; therefore, disease progression in pH water recipients may be gut microbiome-dependent [50]. This is evidenced by the suppression of disease progression, including low autoantibody level and proteinuria in NW recipients who have been given the cecum microbiota of AW recipients orally through microbiota manipulation [30].

*Turicibacter* spp*.,* as a part of the family *Erysipelotrichaceae*, is positively associated with administering acidic water (AW) in the nephritic stage of SNF_1_lupus-prone mice [30]. In MRL/lpr mice, this genus is included in the top ten genera with the highest level and is a biomarker in the GF + SLE group [51, 52].

An increase in *L. reuteri* is also demonstrated in AW-fed nephritic stage SNF1 mice and high tryptophan diet in TC mice [26, 30]. Environmental and genetic influences play a significant role in the response of *L. reuteri*. Despite its potential as a probiotic through its anti-inflammatory and Treg-inducing properties, the increased growth of *L. reuteri* has the potential for translocation, which may induce the pathogenesis of lupus related to organ pathology and pDC/IFN triggering properties [27, 37].

A high tryptophan diet in TC mice can also increase *L. johnsonii*, where these taxa have been thought to increase after developing autoimmunity that can exacerbate lupus. In different lupus-susceptible mouse models, the effect of *Lactobacillus* spp. abundance will also be different as in TLR7.1Tg and TC mice; the more *Lactobacillus* spp., the worse the activity of SLE disease [27]. An increase in the abundance of *Bacteroides dorei, Prevotella*, and *Prevotellaceae* (phylum *Bacteroidetes*) has also occurred in TC mice with a high tryptophan diet [26]. The formation of indole derivative metabolites from tryptophan can be carried out by Gram-negative bacteria such as *Bacteroides* spp*.,* which can negatively affect TC mouse immune cells [26, 53, 54]. On the other hand, tryptophan can be converted to kynurenine by indoleamine 2,3-dioxygenase (IDO) and is regulated by IFN-1, a cytokine that is increased in SLE patients. This increased conversion can lower serotonin levels in serum, leading to a severe SLE phenotype with elevated anti-dsDNA antibodies and nephritis as demonstrated in TC mice [26, 55]. Sources of tryptophan are high-protein foods such as eggs, meat, fish, beans, nuts, and cheese [53].

In pristane-treated Balb/c mice, to demonstrate the phenotypes of *lupus*, administration of tRA at week ten after pristane injection has resulted in an increase in *Bacteroidetes* associated with worsening glomerular pathological scores and proteinuria [29]. In addition, there is also a decrease in *Firmicutes*, which contributed to a decrease in the *Firmicutes*/*Bacteroidetes* ratio, as happened in SLE patients compared to healthy controls [29, 56]. This suggests that the tRA diet can positively and negatively affect SLE activity depending on the administration time. The worsening effect of diet on various microbiomes is depicted in Figure 3.

### 4.3. Diet and Metabolic Function of the Microbiome

In addition to microbial composition, diet can also impact the metabolic function of the microbiome. In the gut microbiome of MRL/lpr mice treated with RA, there is a change in genes in carbohydrate metabolism (glycoxylate, galactose, and dicarboxylate). In contrast, there is a decrease in galactose metabolism and glycolysis and an increase in glycoxylate and dicarboxylate metabolism in lupus disease [14]. The metabolism of amino acids such as histidine, phenylalanine, tyrosine, and tryptophan can also be affected by RA treatment, so these metabolic changes indicate weakening of lupus symptoms [14]. On the other hand, through metagenomic prediction of microbial metabolic function, there is a decrease in genetic information processing and nucleotide metabolism as well as significant increases in membrane transport, signal transduction, cell motility, and sporulation genes which can be exacerbated by VARA treatment and worsening disease [14].

Diet is not the only definite factor that influences the course of SLE disease related to changes in the microbiome. Another factor that must be considered is the use of drugs in SLE patients. The decrease in *Firmicutes* and *Enterococcaceae* occurred with hydroxychloroquine (HCQ), both short-term/high-dose and long-term [57–59]. As an anti-inflammatory and immunosuppressive agent, glucocorticoid therapy is not only capable of reducing cytokine production but also has the potential to stabilize the gut microbiome, which is characterized by a decrease in *Bacteroidetes* with an increase in *Lactobacillus, Streptococcus*, and *Bifidobacterium* [60]. However, in the human study, there has been no further discussion of the effects of antimalarial drugs and non-steroidalanti-inflammatory drugs on microbiome activity.

The limitation of this systematic review is that most research subjects have used animal-prone lupus models with varied types and genders, and only one study has included SLE patients. In addition, all eligible studies did not use uniform tools to measure lupus disease activity. However, SLE development might be seen through lupus disease parameters such as antibody responses, organ pathology, gut leakiness or bacterial translocation, microbiota manipulation or transfer, and change in microbiome metabolic functions. Interestingly, even dysbiosis itself has become a parameter of SLE activity [61]. Moreover, this study only focused on the effect of diet on microbiome activity in SLE patients without considering the current treatment. Further studies exploring factors related to the axis of diet-microbiome-SLE disease activity are of greatly needed. Moreover, considering the drugs interaction involving microbiome in SLE therapy maybe enhance the quality of life of SLE patients.

## 5. Conclusions

The nutrient-gut microbiome relationship has a significant role in the pathology of systemic lupus erythematosus (SLE). Resistant starch (RS) diet from fibrous foods can be fermented by the gut microbiome into several types and amounts of short-chain fatty acids (SCFAs) and have a beneficial effect on SLE outcomes in lupus-prone mice models. Moreover, polyphenols, especially from apples and oranges, can increase beneficial microorganisms in SLE patients. These findings suggested a new therapeutic approach through dietary intervention to treat autoimmune diseases.

## Figures and Tables

**Table 1:** Main characteristics of the studies about diet that affect the gut microbiome related to SLE disease activity.

No	Authors	Study design	Populations	Exposures	Target phyla/genera/families/others	Indicators for assessing the activity of SLE	Results
*Human study*							

1.	Cuervo et al. [25]	Case control	Female SLE patients (*n* = 20)	Dihydrochalcones (from apple)	*Bifidobacterium*	Not informed	Increased microbial diversity in line with polyphenol intake
				Flavanones (95% from oranges and their products)	*Lactobacillus*		
				Flavones (mainly from oranges and their products)	*Blautia*		
*Mice model studies*							

2.	Zhang et al. [14]	Clinical trial	MRL/Mp-Faslpr (MRL/lpr) mice	Retinoic acid (RA) suspended in canola oil as the vehicle; 6 mg RA/kgBW daily from 6 to 14 weeks of age	*Lactobacillaceae*	Lupus disease parameter: lymphadenopathy and glomerulonephritis	Significant improvement of lupus symptoms
					*Erysipelotrichaceae*	Not informed	
				Vitamin A-retinoic acid (VARA) consists of 11.2 mg all-trans-retinyl palmitate (RP)/kgBW was mixed with 0.6 mg RA/kgBB daily from 6 to 14 weeks of age	*Lachnospiraceae*	Lupus disease parameter: lymphadenopathy and glomerulonephritis	Worsened lupus symptoms
					*Rikenellaceae*	Not informed	
					*Erysipelotrichaceae*		Combination with RA decreased Erysipelotrichaceae composition

3.	Johnson et al. [30]	Clinical trial	Female SWR x NZB F1 (SNF1) mice	Neutral water (NW) pH 7.0–7.2	*Rikenellaceae* family	Not informed	In prenephritic stage: positive correlation between Rikenellaceae with NW treatment
					*Pedobacter kwangyangensis* and *Flavobacterium antarcticum*		In prenephritic stage: higher level of *Bacteroidetes* and low ratio of *Firmicutes*/*Bacteroidetes*
				Acidic water (AW) pH 3.0–3.2	*Lactobacillus reuteri and Turicibacter* spp.		In nephritic stage: contributes to higher average *Firmicutes*/*Bacteroidetes* ratio than the NW group
					*Christensenellaceae family*, *Ruminococcus gnavus*, *Peptoniphilus coxii*, *Caloramator mitchellensis, Lactobacillus hayakitensis*, *L. intermedius*, *L. siliginis*, *L. equi*, *Peptoniphilus methioninivorax*, *Trichodesmium hildebrandti*, *Hydrocarboniphaga daqingensis*, *Polaribacter butkevichii*		Positive association between AW diet and microbial diversity

4.	Edwards et al. [28]	Clinical trial	Female MRL/MpJ-Faslpr/J (MRL/lpr) mice	Chow diets (3 until 16 weeks of age):	*Lachnospiraceae*	Not informed	*Lachnospiraceae* higher in 2018 diet compared to RD diet and 7013 diet
				(1) RD diet (open standard diet D11112226 purified-ingredients diet):			
				(i) Casein (undetectable level of isoflavones)			
				(ii) Slightly lower in vitamin A, vitamin K, niacin, and pantothenic acid			
				(iii) Soluble fiber inulin			
				(iv) Higher concentration of carbohydrates			
				(2) 7013 diet (7013 NIH-31 modified 6% mouse/rat sterilizable diet)			
				(i) Fish meal, soybean meal, and alfalfa meal (moderate level of isoflavones)			
				(ii) Mixture of plant-derived soluble and insoluble fibers			
				(3) 2018 diet (Commercial 2018 Teklad Global 18% Protein Rodent Diet):			
				(i) Soybean meal (highest level of isoflavones)			
				(ii) Higher concentrations of vitamin E and B vitamins riboflavin, biotin and pyridoxine–HCl than RD diet and 7013 diet			
				(iii) Mixture of plant-derived soluble and insoluble fibers	*Lactobacillaceae*		Only negligible levels among all diet groups

5.	Zegarra-Ruiz et al. [27]	Clinical trial	Female lupus-prone TLR7.1Tg C57BL/6 (TLR7.1Tg) mice	RS diet (TD. 150492 for SPF and TD. 160604 for GF mice) enriched for hi-maize 260 (40 g/kg) (7 or 11 weeks according to the experimental setup or up to 32 weeks of age or time of death for survival studies)	*Clostridiales*	Integrity of epithelial using FITC-dextran (Growth and translocation)	Positive association between diet and microbial composition. Capable of fermenting RS into SCFAs.
					*L. reuteri*		Inverse correlation between diet and microbial composition. Decreased translocation in RS diet has a positive effect on SLE.

6.	Choi et al. [26]	Clinical trial	Female triple congenic lupus-prone mice (B6.Sle1.Sle2.Sle3) stimulated (TC mice)	High dietary tryptophan (at 6 weeks of age for 4 months)	*Paraprevotella*, *Lactobacillus*, *Prevotellaceae*, and *Bacteroides dorei*	Fecal transfers from TC mice fed tryptophan into GF B6 mice to test functional consequences due to microbial changes	Lupus-associated phenotypes (higher cell number in mesenteric lymph node and serum anti-dsDNA IgM, number of Tfh, Th17, germinal center B cell, and plasma cell)

7.	Abdelhamid et al. [29]	Clinical trial	Pristane-treated Balb/c mice	Daily all-trans-retinoic acid (tRA) diet 1 mg/kg body weight and adjusted according to body weight obtained biweekly from 3 to 9 months of age	*Clostridiales*	Not informed	Increased microbial diversity in line with the treatment at 2 weeks after pristane injection
					*Bacteroidales*	Glomerular pathological score and proteinuria level	Positive correlation between diet, microbial composition, and glomerular pathological scores at 10 weeks after pristane injection
					*Firmicutes*	Not informed	Decreased *Firmicutes*/*Bacteoidetes* ratio, similar in active disease of SLE

## Data Availability

The data used to support the findings of this study are included within the article.
